# The Killing of African Trypanosomes by Ethidium Bromide

**DOI:** 10.1371/journal.ppat.1001226

**Published:** 2010-12-16

**Authors:** Arnab Roy Chowdhury, Rahul Bakshi, Jianyang Wang, Gokben Yildirir, Beiyu Liu, Valeria Pappas-Brown, Gökhan Tolun, Jack D. Griffith, Theresa A. Shapiro, Robert E. Jensen, Paul T. Englund

**Affiliations:** 1 Department of Biological Chemistry, Johns Hopkins Medical School, Baltimore, Maryland, United States of America; 2 Department of Medicine, Johns Hopkins Medical School, Baltimore, Maryland, United States of America; 3 Department of Cell Biology, Johns Hopkins Medical School, Baltimore, Maryland, United States of America; 4 Lineberger Cancer Center, University of North Carolina, Chapel Hill, North Carolina, United States of America; 5 Department of Pharmacology and Molecular Sciences, Johns Hopkins Medical School, Baltimore, Maryland, United States of America; Yale University, United States of America

## Abstract

Introduced in the 1950s, ethidium bromide (EB) is still used as an anti-trypanosomal drug for African cattle although its mechanism of killing has been unclear and controversial. EB has long been known to cause loss of the mitochondrial genome, named kinetoplast DNA (kDNA), a giant network of interlocked minicircles and maxicircles. However, the existence of viable parasites lacking kDNA (dyskinetoplastic) led many to think that kDNA loss could not be the mechanism of killing. When recent studies indicated that kDNA is indeed essential in bloodstream trypanosomes and that dyskinetoplastic cells survive only if they have a compensating mutation in the nuclear genome, we investigated the effect of EB on kDNA and its replication. We here report some remarkable effects of EB. Using EM and other techniques, we found that binding of EB to network minicircles is low, probably because of their association with proteins that prevent helix unwinding. In contrast, covalently-closed minicircles that had been released from the network for replication bind EB extensively, causing them, after isolation, to become highly supertwisted and to develop regions of left-handed Z-DNA (without EB, these circles are fully relaxed). *In vivo*, EB causes helix distortion of free minicircles, preventing replication initiation and resulting in kDNA loss and cell death. Unexpectedly, EB also kills dyskinetoplastic trypanosomes, lacking kDNA, by inhibiting nuclear replication. Since the effect on kDNA occurs at a >10-fold lower EB concentration than that on nuclear DNA, we conclude that minicircle replication initiation is likely EB's most vulnerable target, but the effect on nuclear replication may also contribute to cell killing.

## Author Summary

*Trypanosoma brucei* is a protozoan parasite that causes cattle disease and human sleeping sickness in Africa. Trypanosomes are primitive eukaryotes with atypical biological features. One well-studied example is their mitochondrial genome, known as kinetoplast DNA or kDNA. kDNA, resembling medieval chain mail, is a giant network of interlocked DNA rings known as minicircles and maxicircles. Here we study ethidium bromide, a drug used for over 50 years for treating cattle infected with trypanosomes. EB's killing mechanism has been elusive, and many thought it could not involve kDNA. We now report that EB kills these parasites by blocking the initiation of minicircle replication, and, at higher drug concentration, it also blocks nuclear replication.

## Introduction

*Trypanosoma brucei*, the African trypanosome, is a protozoan parasite causing human sleeping sickness and the disease nagana in cattle. For both humans and livestock, there is a compelling need for less toxic and more effective drugs. One drug, ethidium bromide (EB, known in the veterinary world as homidium; see structure in Fig. S1) was synthesized as a trypanocide over a half-century ago by chemists at Boots Pure Drug Co., Ltd, in Nottingham, U.K. [1]. EB is an intercalating agent [2] widely used as a fluorescent stain for DNA in electrophoresis gels although many scientists are concerned about its mutagenicity. Given its potential dangers, many would be shocked to learn that EB is still used for treating cattle that provide beef and milk for human populations [3].

EB has long been known to promote loss of the trypanosome's mitochondrial genome, the giant DNA network named kinetoplast DNA (kDNA). kDNA is an amazing structure, constituting ∼5% of the cell's total DNA, that consists of several thousand minicircles (each 1 kb) and a few dozen maxicircles (each 23 kb). These circles are interlocked together in one huge planar network that has a topology like that of medieval chain mail (reviewed in [4], [5]). The kDNA network *in vivo* is condensed into a compact disk-shaped structure residing in the mitochondrial matrix. Maxicircles, like conventional mitochondrial DNAs, encode rRNAs and a few mitochondrial proteins (e.g., subunits of respiratory complexes). However, to form a functional mRNA with an open reading frame, most maxicircle transcripts are edited by insertion or deletion of uridylates at specific sites. Minicircles encode guide RNAs that are editing templates (reviewed by [6]).

The unusual characteristics of kDNA and its replication pathway (discussed below), coupled with the lack of DNA networks in mammalian cells, would suggest that kDNA and its replication proteins should be attractive targets for selective chemotherapy. But this possibility was ignored for many years because of the existence of bloodstream form (BSF) trypanosomes lacking kDNA. These dyskinetoplastic (Dk) BSFs appear spontaneously or have been induced by treatment with DNA binding agents such as acriflavin or EB [7], [8], [9]. Their existence suggested that kDNA is not essential for viability of BSFs and therefore would not be a drug target. Acriflavin causes loss of kDNA in other trypanosomatids, such as *Leishmania tarentolae*, but in this case the cell dies because maxicircles encode essential mitochondrial proteins [10].

However, a recent discovery changed our thinking on kDNA as a target for chemotherapy. A report that RNAi knockdown of a mitochondrial RNA ligase involved in editing is lethal to BSFs indicated that editing, and therefore kDNA, are indeed needed for viability [11]. Subsequent studies revealed that an essential maxicircle gene product in BSFs is the A6 subunit of the membrane-embedded F_o_ component of the F_1_ F_o_ ATP synthase [12]. In procyclic trypanosomes this enzyme makes ATP, but in BSFs it couples the reverse reaction, ATP hydrolysis, to generation of a mitochondrial membrane potential required for viability [12], [13]. If editing of A6 is inactivated in either lifecycle stage, then the ATP synthase cannot function and the parasite dies. The reason that some Dk cell lines survive is that they acquire a compensating mutation in a nuclear gene (encoding the gamma subunit of the F_1_ portion of the ATP synthase) that rescues Dk cells and permits their survival [12], [14], [15]. Since the compensating mutation occurs at low frequency, kDNA and proteins involved in its replication or gene expression should be valid drug targets in BSFs.

Since EB-mediated kDNA loss could be due to a block in network replication, we will briefly discuss this pathway (reviewed in [4], [5]), focusing first on minicircles. In the first step, a topoisomerase (topo) II releases monomeric covalently-closed minicircles from the network into the kinetoflagellar zone (KFZ), a region of the mitochondrial matrix between the kDNA disk and membrane near the flagellar basal body. Proteins in the KFZ initiate and propagate unidirectional theta-type replication. Free minicircle progeny probably segregate in the KFZ and then migrate to the antipodal sites, two protein assemblies flanking the kDNA disk and positioned ∼180° apart. Here RNA primers are removed, and most but not all gaps and nicks (we will refer to these discontinuities as gaps) are repaired. The newly replicated minicircles, still containing at least one gap, are then attached to the network periphery by the mitochondrial topoisomerase II (TbTopoII_mt_) positioned in the antipodal sites. Following completion of replication, when the minicircle copy number has doubled, the network splits in two and minicircle gaps are repaired. The progeny kinetoplasts then segregate into the daughter cells during cytokinesis. Much less is known about maxicircle replication. They also replicate unidirectionally as theta structures, but unlike minicircles, they remain linked to the network during replication (see [16], [17], [18] for information on maxicircle replication).

In this paper we report new and unexpected effects of EB on trypanosomes that reveal, for the first time, how this drug kills these parasites.

## Results

### Effect of EB on cell growth and kDNA size

**Figure 1:**
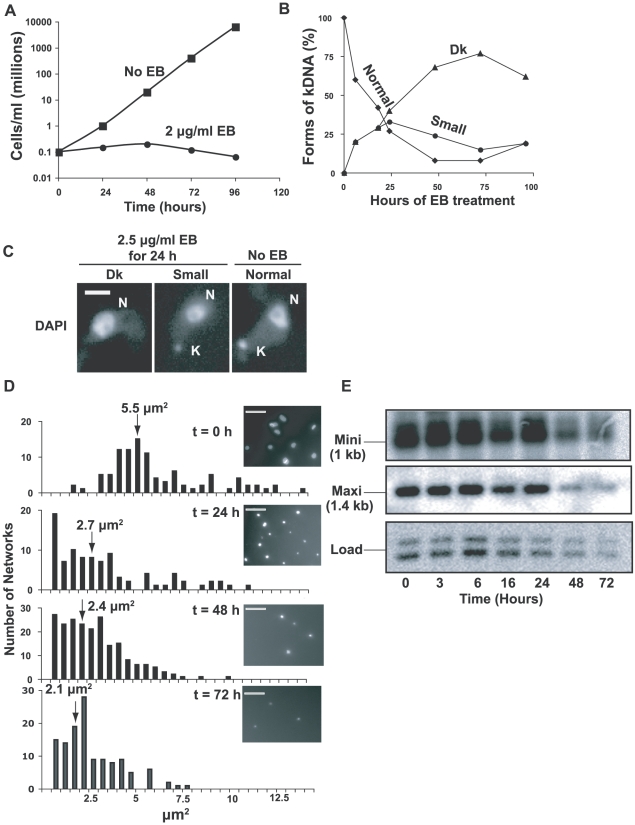
Effects of EB on growth and kDNA. (A) Effect of EB (2 µg/ml) on growth. Values of parasites/ml on y-axis are measured value times dilution factor. (B) Effect of EB on kinetoplast size. DAPI-stained cells (∼100 per time point) were visually categorized by kinetoplast size. Dk, dyskinetoplastic or no detectable kDNA. (C) Fluorescent images of different-sized kinetoplasts seen in DAPI-stained cells untreated or following EB treatment. Scale bar, 5 µm. (D) Bar graphs showing surface areas, calculated with IPLabs software, of DAPI-stained networks isolated from cells treated for the indicated time with 2 µg/ml EB. Arrows show average size. Insets show a representative field at each time point. Scale bar, 5 µm. (E) Time course of kDNA loss. Total DNA digested with *Hind* III/*Xba* I was fractionated on a 1.5% agarose gel (10^6^ cell equivalents/lane). A Southern blot was probed for minicircles (only the 1 kb fragment is shown) and maxicircles (probe recognizes only a 1.4 kb fragment).

We tested EB concentrations ranging from 0.01 µg/ml to 10 µg/ml on growth of BSF *T. brucei* 427. Although 0.02 µg/ml EB stopped growth in 3 days, in most experiments we used 2 µg/ml (5 µM) EB to compare our results with previous data [19]. This concentration arrested growth and killed the cells (Fig. 1A).

We next used fluorescence microscopy of DAPI-stained cells to examine EB's effect on the kinetoplast (Figs. 1B, C). It was not surprising that EB initially caused production of small kinetoplasts, and by 72 h, more than 75% of cells had no detectable kDNA (we refer to these cells as Dk even though we did not confirm that they are completely devoid of kDNA). Examples of cells with normal kinetoplasts, small kinetoplasts, and none at all (Dk) are shown in Fig. 1C. We then isolated and DAPI-stained networks from EB-treated cells and measured their surface areas (Fig. 1D). Networks from untreated cells averaged ∼5.5 µm^2^ (in agreement with previous measurements [20], [21]), but after a 3 day EB treatment had shrunk to an average area of ∼2.1 µm^2^ (Fig. 1D). Because kDNA isolation involves centrifugation, there may have been selective loss of the smallest networks; thus the average area may be smaller than indicated.

To prove that kinetoplast shrinking is due to loss of minicircles and maxicircles, we digested total DNA with *Hin*d III/*Xba* I. After electrophoresis, we probed a Southern blot for maxicircle and minicircle fragments (Fig. 1E). Taking into account the loading control, more than half of the minicircles and maxicircles were lost by 72 h.

### Effect of EB on free minicircles

Covalently-closed circular DNAs isolated from prokaryotic or eukaryotic cells are always negatively supertwisted, with one exception. Remarkably, covalently-closed minicircles in a kDNA network [22] or free replication intermediates [23] are fully relaxed *in vivo*. However, we expected that minicircles isolated from EB-treated cells, either in a network or free, would become negatively supertwisted. This prediction was based on the equation, Lk  =  Tw + Wr, that relates linking number (Lk) to twist (Tw) and writhe (Wr) [24]. Since minicircles *in vivo* are relaxed (Wr  = 0), then Lk  =  Tw, and for a 1 kb minicircle Tw is ∼96 (assuming 10.4 bp/helical turn). EB in the mitochondrial matrix should intercalate into minicircles, thereby reducing their helical twist. Since EB binding does not change Lk, the decrease in twist is compensated by an increase in writhe, or positive supertwisting. A mitochondrial topoisomerase could then remove the positive supertwists, reducing Lk. EB, however, would be still bound to the DNA and would be removed only when DNA is isolated. After EB removal, the twist would revert to its normal level of ∼96, but since Lk had been reduced *in vivo*, the writhe would decrease, producing negative supertwisting.

**Figure 2:**
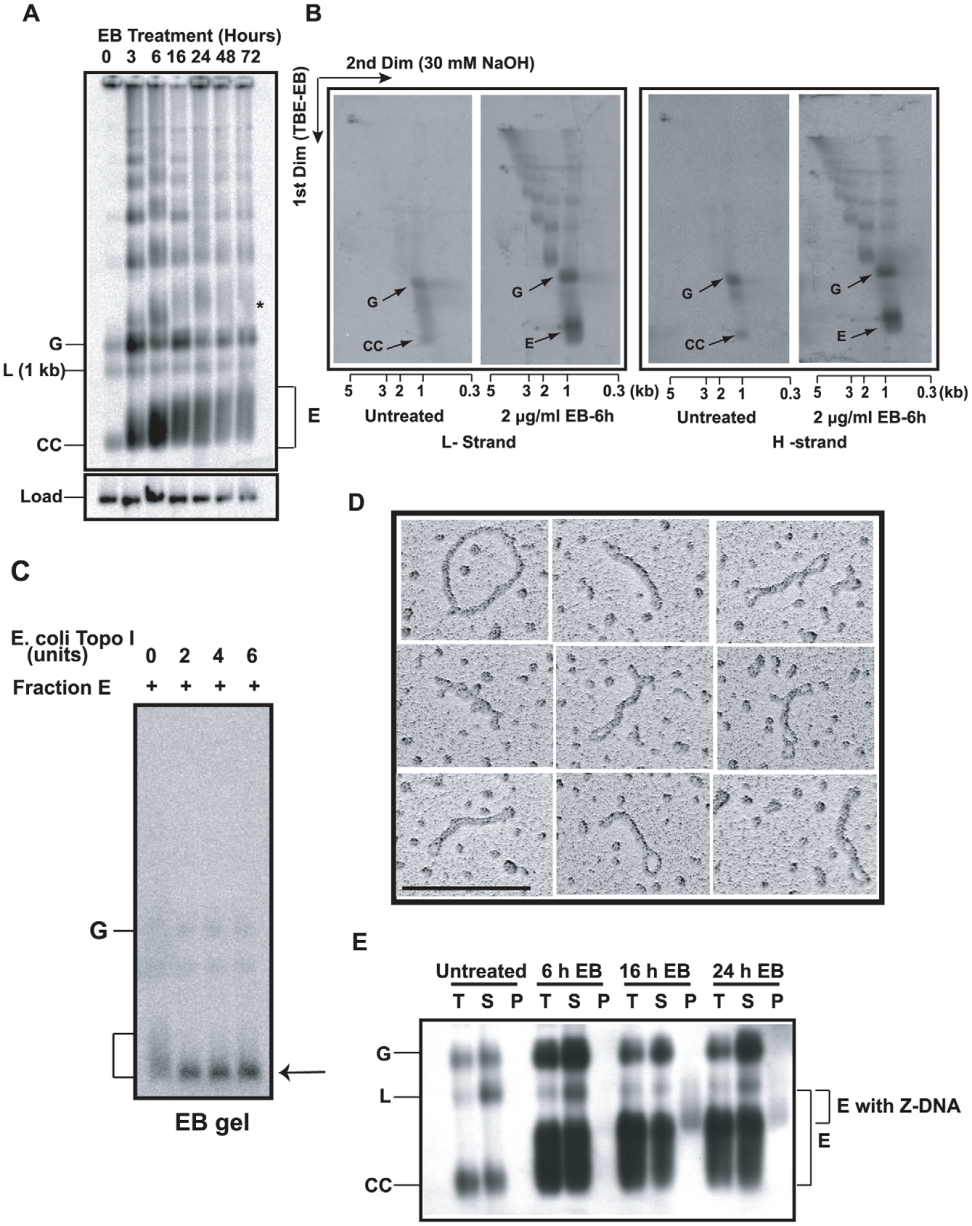
Discovery and characterization of Fraction E. (A) Effect of EB on free minicircle population. Total DNA (5×10^6^ cell equivalents/lane), from cells treated with 2 µg/ml EB for indicated time, was fractionated on an agarose-EB gel. The autoradiograph is a Southern blot probed for minicircles and hexose transporter gene (load). G, gapped, L, linearized, and CC, covalently-closed minicircles. E is Fraction E. * is probably interlocked minicircle dimer forming a smear like that of Fraction E. We usually see only trace amounts of linearized minicircles in untreated cells (0 time point). (B) Two-dimensional neutral-alkaline gel electrophoresis of free minicircles. The sample was total DNA (5×10^6^ cell equivalents/lane), from untreated or EB-treated (6 h, 2 µg/ml) cells. ^32^P-labeled oligonucleotides were used for probing minicircle L (left two panels) and H (right two panels) strands. Scales below gels are sizes of linear markers. Abbreviations of minicircle species are same as in Panel A. See [25], [26] for description of other bands in these gels. (C) Treatment with Topo I. Fraction E (10 µl, sucrose gradient purified) was treated with *E. coli* topo I [51] and fractionated on an agarose-EB gel. Arrow indicates position of covalently-closed relaxed minicircles. (D) EM of fraction E also sucrose gradient purified. Molecule at upper left is a relaxed minicircle. Scale bar, 200 nm. (E) Fraction E contains regions of Z-DNA. Total DNA (T) from 50 ml of culture (0.8×10^6^ cells/ml) of untreated or EB (2 µg/ml)-treated cells was immunoprecipitated with anti-Z DNA antibody and then with protein G-Sepharose [24]. Upon centrifugation, DNA in supernatant (S) and pellet (P) was electrophoresed and probed for minicircles. Increases in linearized minicircles could be due to nuclease contamination of the antibody. Gel conditions and abbreviations of minicircle species are same as in Fig. 2A.

To test the effect of 2 µg/ml EB on free minicircle replication intermediates, we fractionated total DNA from treated cells on an agarose-EB gel that resolves relaxed covalently-closed minicircles (CC; the replication precursors) from gapped minicircles (G, the replication products). A Southern blot (Fig. 2A) revealed a profound increase in a new minicircle species, designated fraction E, that migrated near covalently-closed minicircles. Fraction E forms a smear that broadened between 3 and 72 h, and at later time points its level declined. There was also an increase in multimeric minicircles, and at least one species, probably an interlocked dimer (marked *), formed a smear like that of fraction E. As observed previously [19], EB caused a rise in linearized minicircles (maximum at 1 h, data not shown) that then leveled off. Gapped free minicircles rose during the first 3 h followed by a small decline.

We then set out to identify fraction E, first using a 2-D gel to compare DNA from untreated cells with that from cells treated 6 h with 2 µg/ml EB. The first dimension conditions were the same as those used for the gel in Fig. 2A; the second dimension was run in 30 mM NaOH. This gel system is effective in resolving free minicircle species [25], [26]. We then probed a Southern blot with ^32^P-oligonucleotides of comparable specific radioactivity and complimentary to either the L-strand (Fig. 2B, left panel) or H-strand (Fig. 2B, right panel). From untreated cells, we observed the well-documented resolution of covalently-closed minicircles (CC) and gapped minicircles (G). These two species hybridized equally to both probes because their strands are equimolar. Most importantly, this experiment showed that fraction E consists of minicircles in the 1-kb range that also have roughly equimolar amounts of L and H strands.

In our second approach, we treated fraction E (purified by sucrose gradient sedimentation [25]) with *E. coli* topo I, an enzyme that relaxes negative but not positive supertwists [27]. Electrophoresis on an agarose-EB gel (Fig. 2C) demonstrated that the fraction E smear collapsed into a band that migrated with covalently-closed relaxed monomers.

Finally, EM of Fraction E provided the most compelling evidence on its structure (Fig. 2D), proving that Fraction E is a family of supertwisted free minicircles. The topo I experiment (Fig. 2C) showed that the supertwisting is negative.

### Fraction E contains left-handed Z-DNA helix

In addition to supertwisting, DNA can compensate for severe underwinding by forming single-stranded regions and regions of left-handed helix [28]. In fact, we previously detected Z-DNA in highly supertwisted free minicircles from cells undergoing RNAi of p38, a protein involved in replication initiation [25]. To determine whether a region of Z-DNA forms in free minicircles from EB-treated cells, we immunoprecipitated total DNA with anti-Z DNA antibody, centrifuged the immune-complexes, and fractionated the DNA from the supernatant and pellet on an agarose-EB gel. A Southern blot (Fig. 2E) showed that minicircles from untreated cells or following 6 h of EB treatment had no fraction E in the pellet, indicating that these minicircles contain no Z-DNA. However, after 16 or 24 h of EB exposure, we observed part of fraction E (with the slowest electrophoretic mobility) in the pellet, demonstrating that some fraction E minicircles contain regions of Z-DNA. We conclude that fraction E minicircles have a broad distribution of linking numbers and the most severely underwound have sequences that flip into Z-DNA.

*T. brucei* has 3 known mitochondrial topoisomerases, TbTopoII_mt_ [29], TbTopoIA_mt_ [30], and TbTopoIB (the latter is functional in both nucleus and mitochondrion [31]). In RNAi experiments in Fig. S2, we showed that knockdown of TbTopoII_mt_, but not TbTopoIA_mt_ or TbTopoIB, prevented reduction of the linking number of EB-bound free minicircles and thereby inhibited production of Fraction E. Thus, TbTopoII_mt_ is responsible for supertwisting free minicircles isolated from EB-treated cells.

### Effect of EB on replication of minicircles

**Figure 3:**
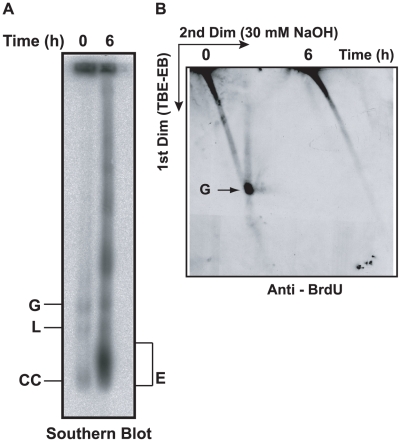
Effect of EB treatment on replication of minicircles. (A) Cells (50 ml, 0.7×10^6^ cells/ml, treated with 2 µg/ml EB for 6 h) were incubated with 50 µM BrdU during the last 40 min. Free minicircles were then fractionated on an agarose-EB gel as in Fig. 2A, and a Southern blot was probed for minicircles. (B) The same amount of DNA used for the gel in Panel A was run on a 2D neutral/alkaline gel (as in Fig. 2B) and a blot was probed with anti-BrdU antibody. BrdU label at top of gel is in nuclear DNA. Abbreviations of minicircle species are same as in Fig. 2A.

Experiments already presented provide strong evidence that EB blocks free minicircle replication. The dramatic rise in covalently-closed monomeric free minicircles in the form of Fraction E (Fig. 2A) indicates that minicircle release from the network occurs in the presence of EB. In addition, their accumulation indicates that the subsequent step, initiation of replication, is inhibited. We therefore assessed replication directly by measuring incorporation of bromodeoxyuridine (BrdU), a thymidine analog, into free minicircles. We incubated log phase cells (±2 µg/ml EB for 6 h) with 50 µM BrdU for the last 40 min. We then isolated total DNA, and fractionated the minicircle species first on a 1-dimensional agarose-EB gel (Fig. 3A, left panel). A Southern blot confirmed that minicircles were more abundant after EB treatment because of the large increase in Fraction E and multimers. To assess BrdU incorporation, we ran the same amount of DNA on a 2-dimensional gel (like that in Fig. 2B). We used this gel to evaluate whether any BrdU-labeled minicircle species accumulated in the presence of EB. Probing a blot with anti-BrdU antibody revealed BrdU incorporation into gapped minicircles in the absence of EB but little or none in any minicircle species during the EB treatment (Fig. 3B). This experiment provided even stronger evidence that EB inhibits initiation of minicircle replication.

### EM of networks isolated from EB-treated cells

**Figure 4:**
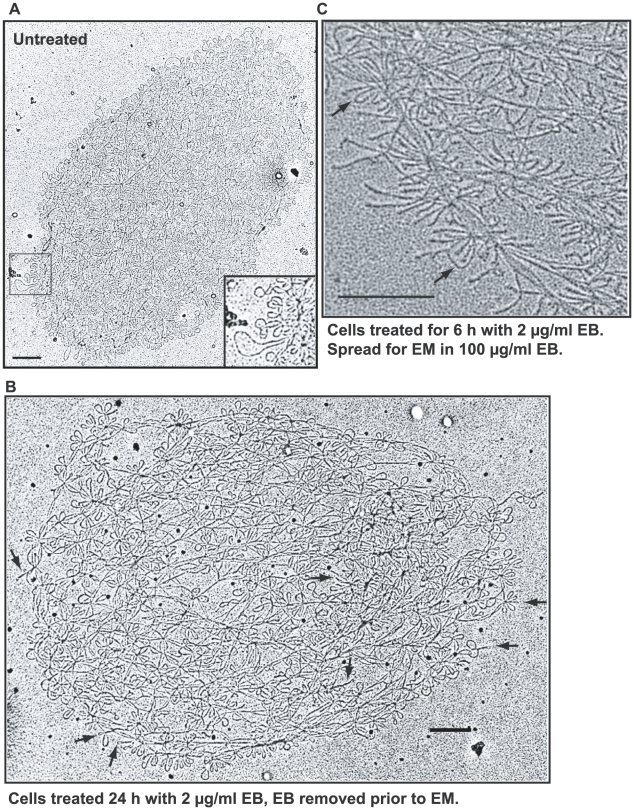
EM of isolated networks. (A) Network from untreated cell spread for EM in the absence of EB. Boxed region is enlarged at lower right. (B) Network from cells treated with EB (2 µg/ml, 24 h), but EB was removed prior to EM. Arrows indicate supertwisted minicircles. (C) Network from EB-treated cells (2 µg/ml EB, 6 h) spread for EM in presence of EB. Prior to EM, EB (100 µg/ml) was added to the DNA, the spreading solution, and the hypophase. Arrows indicate rare untwisted minicircles that likely are nicked. Bars, 500 nm.

Based on our findings with free minicircles, we predicted that minicircles in networks isolated from EB-treated cells would also be negatively supertwisted and we used EM to test this possibility (for this experiment it was essential to extract EB from the DNA prior to EM). We were astonished that most minicircles in networks isolated from cells exposed to 2 µg/ml EB for up to 24 h remained relaxed. Fig. 4B shows a network from a cell treated for 24 h (compare with untreated network in Fig. 4A). Although it is impossible to evaluate many minicircles in the network interior, most on the periphery are relaxed. Only a few (some marked by arrows) appear highly twisted. Fig. S3 shows many more examples of networks from cells treated with EB for 6 h, 15 h, and 24 h. By 24 h, most networks were smaller and more loosely packed (Fig. S3, networks J, K, and L). In these it was easier to evaluate minicircles in the network interior, and most were relaxed. However, one example (Fig. S3, network F) has a substantial region of the network periphery in which most minicircles are twisted.

From the data presented so far, EB treatment of trypanosomes causes severe underwinding of free minicircles but, despite our inability to assess minicircles in the crowded interiors of many networks, it does not have this effect on many and probably most network minicircles. This is remarkable because both DNAs reside in the same cellular compartment, the mitochondrial matrix.

### Testing possible explanations for the absence of supertwisting of network minicircles

One possibility is that the EB caused nicking of network minicircles, thereby preventing supertwisting. To test for nicking, we studied the same networks used in the previous section, but we spread them for EM in the presence of a high concentration of EB (100 µg/ml). If the network minicircles are nicked, they could not supertwist under these conditions. However, as shown by the example in Fig. 4C (others are in Fig. S3, networks H and M), we observed extensive supertwisting of minicircles in most networks. However, some, such as network I in Fig. S3, contained relaxed minicircles. These networks must contain mostly gapped minicircles, and they will be further evaluated below. These data prove that most networks from EB-treated cells contain minicircles that are covalently-closed and that lack of supertwisting is not due to nicking.

A second possibility is that network minicircles bind EB, but there is no topoisomerase available to reduce the minicircle Lk. We showed in Fig. S2 that there is considerable active TbTopoII_mt_ present. Furthermore, we recently reported that TbTopoII_mt_ is present throughout the kDNA disk where it mends holes made by minicircle release during replication [20]. Thus, this possibility is unlikely.

We addressed a third possibility, that network minicircles bind little or no EB, by fluorescence microscopy of EB-treated live cells. We found that brief EB treatment (2 µg/ml added to culture medium) caused distinct staining of the kinetoplast in nearly all cells, but it is possible that some of this staining could be due to free minicircles. Unfortunately, the high motility of trypanosomes precluded capture of adequate images of live cells. Even when the cells were restrained by adherence to poly-lysine coated slides and other methods, they quickly died and lost their staining. Before this happened their localized movement prevented imaging. Instead, Fig. S4 shows images of 16 fluorescent kinetoplasts in formaldehyde-fixed cells. Our conclusion is that kDNA *in vivo* probably binds some EB, but it could be far less than that observed with free minicircles.

**Figure 5:**
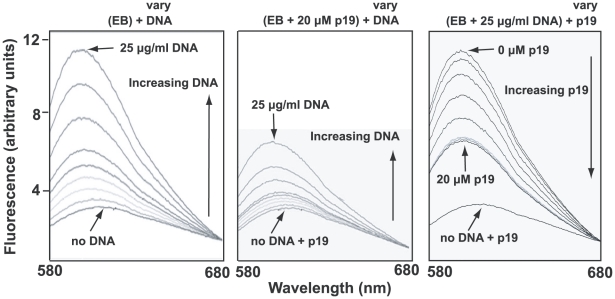
Fluorometric measurement of EB-network binding and the effect of p19. Binding of EB to kDNA was measured by fluorescence spectroscopy using a Fluoromax-3 fluorometer (Horiba). Excitation was at 525 nm and emission was measured from 580 to 680 with a maximum at 604 nm. Titles above each panel show the components of the reaction. Those in parentheses are at constant concentration and those that vary are indicated. Vertical arrows inside each panel show direction of variation. Left panel shows that EB (6.25 µg/ml) emission fluorescence increases markedly on addition of kDNA networks (0, 0.25, 0.5, 1.25, 2.5, 3, 4, 6.5, 25 µg/ml). In middle panel, the same amount of DNA was pre-incubated with 20 µM p19 (room temperature, 5 min) before adding to EB (6.25 µg/ml). Right panel shows displacement of EB (6.25 µg/ml) from DNA (25 µg/ml) by addition of increasing concentrations of p19 (0, 1, 2, 4, 8, 10, 12.5, 15, 20 µM). In this experiment we used *Crithidia fasciculata* networks (purified as described [54]) as they are much easier to isolate than those from *T. brucei*. The preparation of recombinant p19 will be published elsewhere.

A likely reason for low EB binding could be that the condensed network is associated *in vivo* with basic proteins [32], some of which may stabilize the network in its disk-shaped configuration [33]. Such proteins could prevent unwinding of the minicircle helix and therefore would block EB binding. One candidate protein for this role is p19 (GeneDB Accession Number Tb11.02.2420), recently discovered in our laboratory and shown to localize throughout the kDNA disk (unpublished studies of J. Wang, Z. Zhao, B. Liu, P.T. Englund, and R.E. Jensen). Recombinant p19 condenses isolated networks *in vitro* (not shown) to a size comparable to that of the kinetoplast *in situ*. In fluorometric experiments, we observed that network-bound recombinant p19 inhibited EB binding as measured by the marked reduction in EB fluorescence (Fig. 5, compare middle panel with left panel). In a similar assay, p19 could displace EB from a network (Fig. 5, right panel). Thus, the most likely explanation for the absence of supertwisting in many if not most network minicircles is that EB binds poorly to networks *in vivo*. Perhaps those minicircles that do become supertwisted are devoid of proteins, and it is EB binding to these, along with free minicircles, that contributes to the kinetoplast fluorescence observed in Fig. S4.

### Analysis of gaps in networks from EB-treated cells

**Figure 6:**
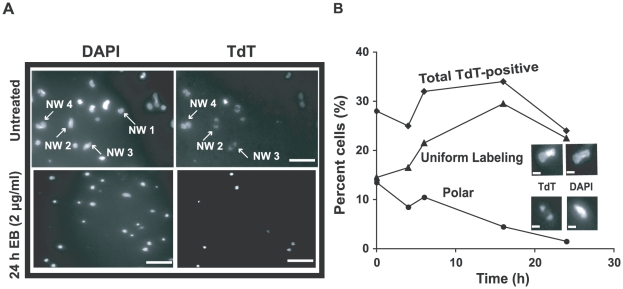
Effect of EB on distribution of gapped circles in isolated networks. Isolated networks were labeled by fluorescein-12-dUTP using terminal deoxynucleotidyl transferase (TdT) [33]. This procedure adds a fluorescent tag to 3′ OH groups flanking minicircle gaps. Because of the relative abundance of minicircles and the distribution of fluorescein fluorescence within networks, most gaps must be in minicircles rather than maxicircles [33]. This procedure reveals the extent of replication of a network. TdT-positive networks are mostly undergoing replication and some are post-replication with gaps yet to be repaired. In wild type cells, the labeling pattern is usually polar or uniform, representing early and late stages of replication respectively. (A) Fluorescent images of networks isolated from untreated cells stained with 2 µg/ml DAPI (upper-left panel) and labeled with TdT (upper-right). Lower panels are the same except cells were EB-treated (2 µg/ml, 24 h). In upper panel, network (NW) 1 is a TdT-negative pre-replication or post-replication network because it stains with DAPI but not TdT. Networks 2 and 3 are replicating networks with polar labeling. Network 4 is TdT-positive with uniform labeling, appearing double-size and ready to divide. Scale bars, 5 µm. (B) Kinetics of change in TdT labeling pattern during EB treatment. Upper inset shows a uniformly labeled network and lower inset shows one with polar labeling. At least 120 networks were counted at each time point. Scale bars, 1 µm.

As mentioned above, some networks from EB-treated cells, examined by EM in the presence of 100 µg/ml EB, had many minicircles that did not twist, suggesting that these minicircles were gapped (an example is network I in Fig. S3). We further investigated these networks by *in vitro* labeling the 3′-OH groups at gaps with terminal deoxynucleotidyl transferase (TdT) and fluorescein-dUTP [34]. In networks from cells untreated with EB, we detected the well-characterized polar labeling of partly replicated networks [34], [35], [36]; labeling is polar because gapped minicircles are attached to the network adjacent to the antipodal sites. From these cells, never exposed to EB (Fig. 6A), 28% of networks were stained by TdT (TdT-positive). This value changed little during 24 h of EB treatment (see time course in Fig. 6B). However, EB dramatically changed the labeling pattern, causing an increase in uniformly labeled networks and a steady decline in polar labeling. The latter suggested that reattachment of newly-replicated minicircles to the network periphery must be strongly reduced by EB, as expected from the fact that EB blocks minicircle replication. In the Discussion we will comment further on these data.

### The effect of ethidium bromide on dyskinetoplastic trypanosomes

**Figure 7:**
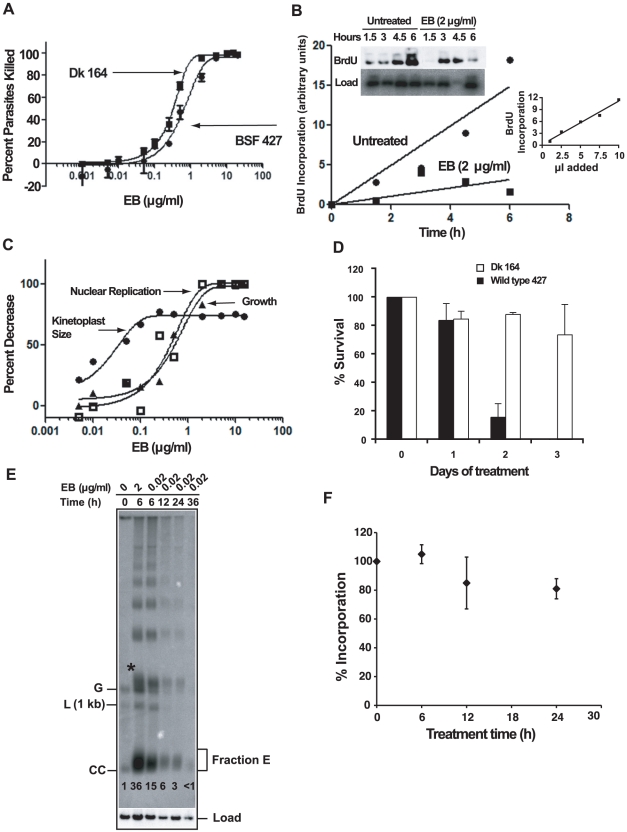
The mechanism of killing BSF trypanosomes by EB. (A) Effect of EB concentration on killing of wild type 427 and Dk 164 trypanosomes. In a 24 h acid phosphatase-based cytotoxicity assay [38], A_405_ was measured on a microtiter plate reader (Molecular Devices). Each EB concentration in an experiment was assayed in quadruplicate, and error bars indicate standard deviations for three experiments. Data were fit to the equation for the sigmoidal E_max_ model [55] using GraphPad Prism software that generated EC_50_ values of 0.30 µg/ml for Dk cells (R^2^ = 0.99) and 0.65 µg/ml for wild type (R^2^ = 0.97). (B) Effect of EB on BrdU incorporation into nuclear DNA of wild type BSF trypanosomes. Cells (10^5^ cells/ml, 10 ml) were incubated with EB (2 µg/ml) and BrdU for up to 6 h. Total DNA at each time point was isolated, fractionated by agarose gel electrophoresis, transferred to a PVDF membrane, probed with anti-BrdU, and labeled DNA was detected by chemiluminescence (quantitated by Image J software; http://rsbweb.nih.gov/ij/). After normalizing for loading using the hexose transporter probe, label in the nuclear DNA band was quantitated by a phosphorimager. Inset graph shows that detection of BrdU was in the linear range (in this experiment, 1, 2.5, 5, 7.5, and 10 µl of the untreated 6 h sample was processed and quantitated with Image J. (C) Effect of EB concentration on kinetoplast size, nuclear replication, and growth of wild type cells. Cells (10^5^ cells/ml, 10 ml) were incubated with the indicated concentrations of EB for 24 h and BrdU was added for the last 2 h. Cytotoxicity (killing) was assayed with 200 µl samples as in Panel A. To measure effect of EB on kinetoplast size, cultures (1 ml) were collected, fixed, stained with DAPI and evaluated by fluorescence microscopy. With regard to kinetoplast size, the reason that some cells retain some kDNA at high EB concentration is not clear. To measure the effect of EB on nuclear DNA replication, total DNA was isolated from remaining samples for electrophoresis as in Fig. 4A; a blot was probed with anti-BrdU and quantitated as in Panel B. Data were fit to the equation for the sigmoidal E_max_ model as in Panel A. (D) Survival of wild type and Dk trypanosomes in 0.02 µg/ml EB. Cells (5×10^4^/ml) were incubated with 0.02 µg/ml EB and at indicated times were counted by hemocytometer (mean ± standard deviation of 3 independent experiments). Cell densities were maintained between 5×10^4^/ml and 10^6^/ml. (E) Comparison of the effects of EB concentration on free minicircles. The experiment was identical to that in Fig. 2A except that EB was either 2 µg/ml or 0.02 µg/ml. Abbreviations and * are same as in Fig, 2A. This experiment was run 3 times with virtually identical results. The fraction E smear in the second lane is narrower than that in Fig. 2A for unknown reasons. The numbers in each lane below fraction E are relative band intensities of fraction E plus covalently-closed minicircles determined by phosphorimaging and corrected for background and load. (F) Effect of 0.02 µg/ml EB on nuclear DNA replication. A culture was treated with EB and at indicated times, cells (200 µl, 2×10^6^/ml) were incubated with [^3^H]thymidine (Perkin-Elmer, 300 µCi/ml, 20 Ci/mmole) in thymidine-free HMI-9 for 2 h (the serum may contain low levels of thymidine). At the end of the labeling, radioactivity was measured in 5% TCA-precipitable DNA. Values on Y-axis are percent of incorporation into untreated cells. The latter incorporated 10.5×10^3^±2.8×10^3^ dpm in 2 h. Roughly 5% of the zero time incorporation should be in kDNA. Each experiment was run with duplicate samples, and plotted values are Mean ± S.D. of three independent experiments.

If EB kills trypanosomes by blocking initiation of replication of free minicircles, then it would be logical to predict that Dk trypanosomes would be resistant to EB killing. We therefore studied the effect of EB on Dk 164, a BSF trypanosome lacking kDNA [8], [37]. Our cytotoxicity assay, using a range of EB concentrations, involved colorimetric measurement of acid phosphatase activity released by lysing living cells after 24 h in wells of a 96-well plate [38]. To our great surprise, we found that EB efficiently killed Dk parasites, and its EC_50_, 0.3 µg/ml, was slightly lower that of the wild type 427 strain, 0.65 µg/ml (Fig. 7A). Thus, EB must have an efficient killing mechanism unrelated to kDNA.

### The effect of ethidium bromide on nuclear DNA replication

A likely possibility for the killing of Dk trypanosomes was that EB also targets nuclear DNA replication. We therefore incubated 427 BSFs with BrdU in the presence and absence of EB for 6 h. Incorporation of this thymidine analog into nuclear DNA increased with time in untreated cells and was inhibited substantially with 2 µg/ml EB (Fig. 7B).

### kDNA is the major target of EB

At first glance, the data in Fig. 7A suggested that a killing mechanism targeting nuclear replication may be as important, or possibly more important, that that targeting kDNA. However, we then considered the possibility that our 24 h cytotoxicity assay may not reveal the effects of EB-mediated kDNA loss in wild type 427 cells. The reason is that the killing would not occur until essential maxicircle gene products were depleted, which, depending on their half-lives, may take a few days. Thus, the 427 cells might contain a time bomb that would not explode until after termination of the 24 h cytotoxicity assay. To evaluate this possibility, we conducted a new cytotoxicity assay using 427 trypanosomes (Fig. 7C). In this assay, we collected cells not only for evaluation of the effect of EB on growth, but also for measurement of kinetoplast size by DAPI staining. In addition, we measured nuclear replication by adding BrdU to the wells for the last 2 h of the 24 h assay. As shown in Fig. 7C, inhibition of nuclear replication occurred at the same EB concentration as the growth effect; thus, inhibiting nuclear replication kills with little delay. In contrast, kDNA loss occurs at a >10-fold lower EB concentration and this loss will ultimately lead to cell death but not during the 24 h cytotoxicity assay. To further evaluate this possibility, we chose an EB concentration, 0.02 µg/ml, that, based on the graphs in Fig. 7C, should eventually kill 427 trypanosomes by targeting kDNA and not kill DK-164 cells which would require a higher EB concentration to inhibit nuclear replication. As predicted, there was little effect of EB on Dk-164 cells (Fig. 7D). Also, as expected, there was little effect on 427 cells during the first 24 h (the duration of the cytotoxicity assay), but survivors were reduced by 75% at day 2 and few cells were alive at day 3. We also noted in our initial studies mentioned in the first paragraph of Results that 0.02 µg/ml EB killed trypanosomes within 3 days.

Most studies in this paper on the effect of EB were conducted at 2 µg/ml. Now it was essential to evaluate further the effects of 0.02 µg/ml EB on minicircle and nuclear DNA replication. For minicircles we conducted an analysis of free minicircles to determine whether covalently-closed free minicircles (including Fraction E) accumulate (because they are unable to initiate replication). In a control experiment, similar to that in Fig. 2A, we found that 6 h treatment with 2 µg/ml EB caused a 36-fold increase in covalently-closed minicircles (Fig. 7E). Reducing the EB concentration 100-fold to 0.02 µg/ml, increased the covalently-closed minicircles 15-fold, still a very potent inhibition of replication. As with 2 µg/ml, the maximum effect of 0.02 µg/ml was at 6 h and at both concentrations there was a large increase in minicircle oligomers. It was not surprising that 0.02 µg/ml EB had a smaller effect in reducing the free minicircle linking number; thus its fraction E smear is more compact. To measure the effect of 0.02 µg/ml EB on nuclear replication, we treated a culture for 0, 6, 12, and 24 h. At each time point we incubated a sample with [^3^H]thymidine for 2 h and then measured acid-insoluble radioactivity (Fig. 7F). Since kDNA constitutes only about 5% of the total DNA, nearly all of the incorporation is in nuclear DNA. These results show that 0.02 µg/ml EB has no effect on nuclear replication at 6 h and a small effect thereafter.

All the experiments in Fig. 7 provide strong evidence that the most sensitive mechanism by which EB kills trypanosomes is to block minicircle replication initiation. However, inhibiting nuclear replication also contributes to the trypanocidal activity.

## Discussion

### The differing effects of EB on network bound minicircles and free minicircles

Our goal in these studies was to determine whether EB kills trypanosomes by its action on kDNA and, if it does, to determine the killing mechanism. One remarkable and unexpected effect of EB treatment was that it caused profound supertwisting of free minicircles (Fig. 2), whereas many if not most network minicircles, in the same cellular compartment, remained relaxed (Figs. 4, S3). The most likely explanation is that the EB binding to the network, although detectable by fluorescence microscopy (Fig. S4), occurs at a much lower level than it does to free minicircles. The large reduction in EB binding to network minicircles is most likely explained by the fact that basic proteins like p19 condense the network *in vivo* and by preventing helix unwinding, inhibit EB binding (Fig. 5). The conclusions from this experiment are limited because they are based on only one protein, p19, whose function is unknown, and because the kDNA disk *in vivo* is likely bound and stabilized by multiple proteins [32]. However, the p19 experiments clearly demonstrate that such proteins can reduce EB binding *in vivo* (Fig. 5). Polyamines also bind DNA and prevent EB binding [39], but proteins seem more likely to be compartmentalized, binding to networks and not free minicircles.

Our EM data provides some clues into the replication status of maxicircles. Gel electrophoresis of topo II-decatenated networks [40] from cells treated with 2 µg/ml EB for 6 to 24 h revealed that 20–50% of maxicircles were covalently-closed (data not shown). Furthermore, in 121 EM images of networks (from cells treated similarly with EB and then observed by EM after EB removal), we observed no supertwisted maxicircles. Thus, the covalently-closed network-bound maxicircles, like most network minicircles, must bind little if any EB *in vivo*. We speculate that the lack of EB binding to maxicircles, as in minicircles, is due to bound protein. If EB does not bind to the maxicircles, it is possible that it has no effect on maxicircle replication. Further studies are needed to address this issue.

### EB inhibits minicircle replication

Our most significant finding was that the most effective mechanism by which EB kills trypanosomes is by inhibition of initiation of free minicircle replication. We initially based this conclusion on the dramatic accumulation of covalently-closed free minicircles, the substrates for replication (Figs. 2A (using 2 µg/ml EB) and 7E (using 0.02 µg/ml EB)). Especially in the case of 2 µg/ml EB, the high level of EB that bound these molecules *in vivo* not only resulted in the development of extensive negative supertwisting after isolation but also, by distorting their helix *in vivo*, must have prevented assembly of replication proteins at the origin. The inhibition of BrdU incorporation into gapped minicircles confirmed the replication block (Fig. 3). Since newly replicated free minicircles attach to the network poles adjacent to the antipodal sites, the EB-mediated decline in networks with polar TdT labeling led to the same conclusion (Fig. 6). But why does the number of networks uniformly-labeled with TdT increase during EB treatment? The likely reason is that a partially-replicated network contains gapped minicircles (labeled by TdT) in the two polar regions and covalently-closed minicircles (not yet replicated and unlabeled by TdT) in the central region [34], [35]. In the presence of EB, few if any minicircles attach to the network poles, preventing enlargement of the polar regions. However, Fig. 2A shows that covalently-closed minicircle release continues, and these must derive from the central region of the network. Once all are released, the two polar regions, containing gapped minicircles, then fuse. Thus the network appears, after TdT labeling, to be uniformly-labeled and small in size. As the network shrinks, minicircle release may become less precise, resulting in release of multimers (Fig. 2A).

We previously found that EB poisons mitochondrial TbTopoII_mt_ (but not nuclear topo II) and that cell lysis by SDS-proteinase K results in linearization of ∼2% of the minicircles [19]. This inhibition of TbTopoII_mt_ is unlikely responsible for cell death because only a small fraction of the TbTopoII_mt_ is trapped as a cleavable complex (for comparison, a more powerful topo II poison, etoposide, causes linearization of 12% [41]). Furthermore, the fact that TbTopoII_mt_ is responsible for the EB-mediated reduction in free minicircle linking number (Fig. S2) proves that much of this enzyme is catalytically active in the presence of EB.

### How EB kills trypanosomes

Since EB blocks initiation of replication of free minicircles, we were initially surprised, and troubled, to find that Dk cells were also killed by EB. However, further study showed that the EB-susceptibility of Dk cells could be explained by inhibition of nuclear DNA replication (Fig. 7B). Furthermore, the effect of EB on kinetoplast size occurred at a >10-fold lower concentration, indicating that kDNA replication is the primary target for EB killing of wild type trypanosomes. Our realization that killing of wild type cells by low EB concentrations does not occur during our 24 h cytotoxicity assays fully clarified this mechanism. The delay in killing must be due to the time needed for the cell to run out of its essential maxicircle gene product, the A6 subunit of the ATP synthase [12]. When we analyzed free minicircles from cells treated 6 h with 0.02 µg/ml EB, we confirmed that this low concentration also inhibits free minicircle replication (Fig. 7E).

Although our cytotoxicity assays suggest that EB acts primarily on kDNA, this drug has at least some effect on nuclear DNA replication. The effect is modest at low concentrations (0.02 µg/ml EB; see Figs. 7C and 7F), but inhibition of nuclear replication becomes significant at higher concentrations (2 µg/ml EB; see Fig. 7C and compare Fig. 7B with 7F). Therefore, the contribution of inhibition of nuclear DNA replication to trypanosome killing depends on the level of EB in the treated animal. The blood concentration of ethidium in cattle injected intra-muscularly with the standard dose of 1 mg/kg is 0.070–0.268 µg/ml at 15 min and ∼0.075 µg/ml after 24 h [42], [43], [44]. These concentrations are for the free base form of ethidium. At the highest concentration detected, 0.268 µg/ml ethidium base (equivalent to 0.34 µg/ml EB), which is transient, inhibition of nuclear DNA replication is only about 40% of maximum effectiveness (see Fig. 7C). The 24 h level, ∼0.075 µg/ml (equivalent to 0.095 µg/ml EB), is about 5 times higher than the 0.02 µg/ml EB used in our experiments, suggesting that inhibition of nuclear DNA replication could contribute to killing. One problem with comparing drug activity *in vitro* to that in animals is that many drugs bind tightly to serum proteins, thereby reducing markedly in animals the concentration of free active drug [42]. We could find no reports on the extent of EB binding to serum proteins, but if high this could substantially reduce the levels of free EB in animals.

One reason why EB may target minicircle replication so effectively is that the intra-mitochondrial EB concentration could be higher than the extracellular concentration, due to a membrane potential-driven transport of ethidium into the mitochondrial matrix [43]. We think it unlikely, but cannot rule out the possibility, that non-specific effects of EB, such as binding to RNA or membranes, could contribute to the killing of trypanosomes.

Another anti-trypanosomal drug still used for treating African cattle is isometamidium chloride [3], an EB derivative (see structure in Fig. S1). In a previous study of whether isometamidium could target kDNA, the effect of the drug on wild type and Dk trypanosomes was investigated using *in vitro* assays [44]. As we found with EB, those authors reported a slightly lower EC_50_ for the Dk cells and concluded that an effect on the kinetoplast could not be the primary target of isometamidium. As with EB, it is possible that an effect on maxicircle gene products was not manifested during their cytotoxicity assays even though theirs were longer than ours. Thus, like EB, the primary target of isometamidium could be minicircle replication.

In conclusion, these experiments prove decisively that low concentrations of EB kill trypanosomes by blocking initiation of minicircle replication. However, it is likely that curing of trypanosome infections depends in part on the effect of EB on nuclear replication. Although it has been shown previously that kDNA is essential for viability of BSF parasite [11], our studies emphasize that enzymes involved in kDNA replication could be valid drug targets for chemotherapy for BSF trypanosomes, not only for those causing bovine trypanosomiasis but also for the parasites causing human sleeping sickness.

## Materials and Methods

### Trypanosome strains, culturing, and EB treatment

All experiments except those involving RNAi or the dyskinetoplastic Dk 164 strain (gift from Ken Stuart, Seattle Biomedical Research Institute) were conducted on BSF *T. brucei* wild-type 427 strain (gift from George Cross, Rockefeller University), cultured as described [45], [46]. For RNAi we used procyclic *T. brucei* 29–13 [47] (also from George Cross) cultured in SDM-79 medium [48] containing 15% fetal bovine serum. Ethidium bromide (Bio-Rad, 10 mg/ml) was kept at room temperature in the dark and added to a final concentration of 2 µg/ml unless otherwise indicated.

### Purification of Fraction E and networks

Total DNA, (purified as described [49]) from 7×10^6^ EB-treated (6 h, 2 µg/ml EB) cells was centrifuged on a 33 ml 5–20% sucrose gradient (Beckman SW28 rotor, 25,000 rpm, 20 h, 4°C) [24]. Aliquots (10 µl) of 1 ml fractions were electrophoresed as in Fig. 2A and a blot probed for minicircles. Fraction E was detected in tubes 9 to 15 and the pool was dialyzed overnight against 25 mM Tris-HCl, pH 8.0, 50 mM NaCl, 10 mM MgCl_2_ (final volume, 9 ml). It was used for *E. coli* topo 1 treatment (Fig. 2C) and EM (Fig. 2D). Networks were isolated as described [50].

### EM

Fraction E was prepared for EM using the cytochrome c drop spreading method [51]. After shadowing, samples were examined in an FEI (Hillsboro, OR) Tecnai 12 TEM and images recorded using a Gatan Inc. (Pleasanton, CA) Ultrascan400 CCD camera.

Isolated kDNA networks were spread on grids using the formamide method [52]. Since many experiments required removal of EB from networks prior to EM, we extracted 6 times with an equal volume of water-saturated *n*-butanol. However, we observed identical results if we did not extract; in that case EB must have been removed by formamide or by ethanol-washing of the grids. After rotary shadowing, networks were photographed using a Hitachi 7600 transmission electron microscope and a DVC 1412M-FW digital camera with AMT Image Capture Engine Software. EM images were uniformly adjusted for brightness and contrast using Adobe Photoshop.

### Gel electrophoresis

Undigested total DNA was electrophoresed to analyze free minicircles, and *Hind*III/*Xba*I digests were electrophoresed to analyze for total minicircles and maxicircles [25]. Both were run in a 1.5% agarose gel in 1X Tris-borate-EDTA (TBE) buffer containing 0.5 µg/ml EB (18 h, 70 V) [41]. DNA was detected by probing Southern blots, and the hexose transporter gene was the load control. For 2-D electrophoresis [25], the first dimension used conditions just described and the second was run in 30 mM NaOH. After electrophoresis, DNA was transferred to GeneScreen plus membrane (Perkin Elmer) and probed with strand-specific 5′-[^32^P]oligonucleotides [25].

### Metabolic labeling with BrdU

Untreated and 6 h EB-treated cells (50 ml, 0.7×10^6^ cells/ml) were pulse-labeled for 40 min with 50 µM bromodeoxyuridine (BrdU) and 50 µM 2′-deoxycytidine [53]. The latter was added to overcome potential allosteric inhibition of ribonucletotide reductase by high concentrations of BrdU. Total DNA was isolated, electrophoresed on either a one-or-two dimensional gel followed by transfer to a nitrocellulose membrane (Schleicher and Schuell). Membranes were blocked, probed with anti-BrdU antibody, and BrdU level was detected by chemiluminescence [25].

## Supporting Information

Figure S1 Three anti-trypanosomal drugs currently used to treat or prevent trypanosome infections in cattle. Ethidium (also known as homidium), isometamidium (also known as samorin), and berenil were first synthesized for their anti-trypanosomal activity [56]. Ethidium, one of the most thoroughly studied DNA binding agents, is a classical intercalator whose planar aromatic ring system inserts between the base pairs in a double helix [2]). Berenil is a symmetrical biguanide that binds DNA's minor groove [57]. Isometamidium incorporates structural features of both ethidium and berenil; its mode of binding to DNA is not well described but it may be a threading-type intercalator [58], in which the ethidium nucleus stacks between base pairs and the berenil-like side chain interacts with the minor groove. Another fluorescent dye used for staining DNA is 4′,6-diamidino-2-phenylindole (DAPI); this compound was also synthesized as a trypanocide [59], [60] but, unlike EB, it is no longer used for that purpose.
(0.08 MB PDF)

Figure S2 TbTopoII_mt_ is the enzyme that decreases the linking number of EB-bound free minicircles in vivo. We investigated which of the 3 known mitochondrial topoisomerases, TbTopoII_mt_ [29], TbTopoIA_mt_ [30], or TbTopoIB (functional in both nucleus and mitochondrion [31]) was responsible for reducing the linking number of EB-bound free minicircles. In this experiment we used procyclic parasites (this lifecycle stage dwells in the tsetse vector's midgut and is conveniently cultured in the laboratory) as RNAi of these 3 enzymes had been well characterized in that stage [49], [30], [31]. (A) RNAi of TbTopoII_mt_. Like EB-treated BSF forms, procyclic cell line 29–13 carrying the TbTopoII_mt_ stem-loop RNAi vector but not induced by tetracycline, was susceptible to EB (2 µg/ml) and accumulated fraction E over the 24 h time course (left panel). Also, the effect of TbTopoII_mt_ RNAi on free minicircles was similar to that reported previously [49] (not shown). Furthermore, a northern blot confirmed that RNAi caused a nearly complete loss of the TbTopoII_mt_ mRNA (right panel, next to bottom). For the northern blot, purified total RNA was fractionated on a 1.5% agarose−7% formaldehyde gel [49] and a blot was probed for TbTopoII_mt_ mRNA. The critical experiment is in the main right panel. In this experiment, RNAi of TbTopoII_mt_ was induced by 1 µg/ml tetracycline. Each day, EB (2 µg/ml), was added to culture for 6 h (at day 0, EB was added at the time of induction with tetracycline). Left-most lane shows that EB causes accumulation of fraction E in absence of RNAi. The rest of the right panel showed that knockdown of TbTopoII_mt_ blocked appearance of fraction E. (B) RNAi of TbTopoIA_mt_ (left panel) and TbTopoIB (right panel). The experimental strategy was the same as in Panel A and the efficacy of RNAi was shown by the effect on the growth curves, which were comparable to published results [30], [31]. In control experiments (not shown), EB (2 µg/ml, 6 h) added to uninduced cells led to production of Fraction E. When RNAi was induced by tetracycline (1 µg/ml), EB (2 µg/ml) was added for 6 h after 0–6 days of RNAi induction (for example, at day 0, EB was added at the time of RNAi induction with tetracycline). Samples were electrophoresed, blotted, probed, and autoradiographed as in Figs. 2A. Since there was no effect on production of Fraction E, we conclude that these enzymes cannot reduce the linking number of EB-bound free minicircles in vivo. This result was expected for TbTopoIA_mt_ as enzymes of this type relax negative but not positive supertwists [27]. Therefore, we conclude that only TbTopoII_mt_ can reduce the linking number of EB-bound free minicircles in vivo and is responsible for production of Fraction E.
(1.15 MB PDF)

Figure S3 EMs of kDNA networks isolated from cells treated with 2 µg/ml EB. Networks A–D were treated in cells for 6 h, networks E–I were treated in cells for 15 h, and networks J–M were treated in cells for 24 h. Unless otherwise indicated, EB was removed prior to EM. Large loops extending from network edge are maxicircles. Small loops are minicircles. Most of the latter are relaxed, but a few, some of which are marked by arrows, are supertwisted. In crowded networks, especially those present after short treatments with EB, it is usually impossible to determine whether minicircles in the network interior are twisted. Network F has a region on the periphery (spanned by the bracket) in which many neighboring minicircles are twisted. Large arrows in panels E and L indicate maxicircle bubbles. Because the strands within the bubble are thinner than the surrounding strands, the bubbles are probably not due to replication but instead are due to denaturation caused by the high formamide concentrations (up to 40%) during spreading for EM. This is not surprising because maxicircles have AT contents of 76.7%. The presence of two bubbles in a maxicircle in panel D provides another strong argument against the possibility that these bubbles are caused by replication. Networks H, I, and M were spread in the presence of 100 µg/ml EB. Most networks in these preparations resemble networks H and M, having covalently-closed minicircles that become supertwisted by EB in vitro. In contrast, a small number, like network I, have most minicircles relaxed, indicating that they contain nicks or gaps; these networks are further evaluated in Fig. 6. In networks from cells treated with EB for 24 h (networks J–M), some had mostly minicircles (J and M) and some had mostly maxicircles (K and L). Analysis of 24 randomly-selected networks from this preparation indicated that 4 were rich in maxicircles, 11 were rich in minicircles, and 9 were intermediate. Scale bars, 0.5 µm.
(6.36 MB PDF)

Figure S4 EB staining of *T. brucei* BSF cells. Log-phase cells were fixed with 4% formaldehyde, washed with PBS-glycine, stained with 2 µg/ml EB for 1 min, and then examined by phase (upper panel) and fluorescence (lower panel) microscopy. Scale bar, 2 µm. Arrows point to kinetoplasts weakly stained by EB. The kinetoplast in the lower left is undergoing division. The larger EB-staining structures are nuclei.
(0.23 MB PDF)
