# Supplementary figures and images for: The Killing of African Trypanosomes by Ethidium Bromide
Source: PLoS Pathog. 2010 Dec 16;6(12):e1001226. doi: 10.1371/journal.ppat.1001226 (PMC3002999; doi:10.1371/journal.ppat.1001226)

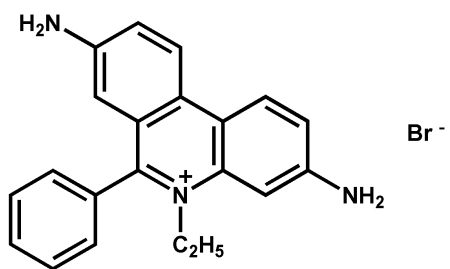

**Ethidium bromide**

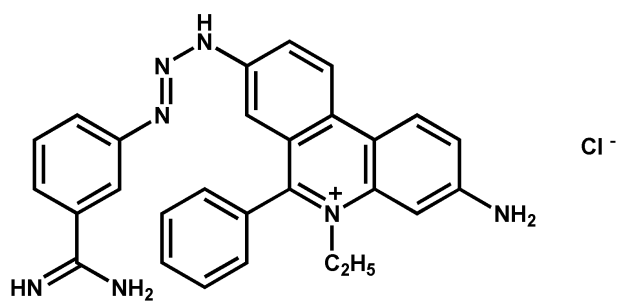

**Isometamedium chloride**

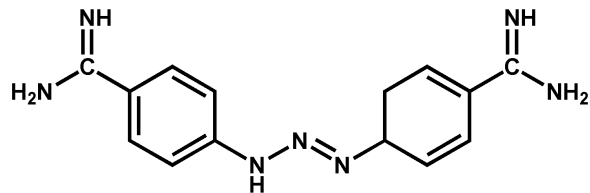

**Berenil**

Supplement: Figure S1 — Three anti-trypanosomal drugs currently used to treat or prevent trypanosome infections in cattle. Ethidium (also known as homidium), isometamidium (also known as samorin), and berenil were first synthesized for their anti-trypanosomal activity [56]. Ethidium, one of the most thoroughly studied DNA binding agents, is a classical intercalator whose planar aromatic ring system inserts between the base pairs in a double helix [2]). Berenil is a symmetrical biguanide that binds DNA's minor groove [57]. Isometamidium incorporates structural features of both ethidium and berenil; its mode of binding to DNA is not well described but it may be a threading-type intercalator [58], in which the ethidium nucleus stacks between base pairs and the berenil-like side chain interacts with the minor groove. Another fluorescent dye used for staining DNA is 4′,6-diamidino-2-phenylindole (DAPI); this compound was also synthesized as a trypanocide [59], [60] but, unlike EB, it is no longer used for that purpose. (0.08 MB PDF) [file ppat.1001226.s001.pdf]

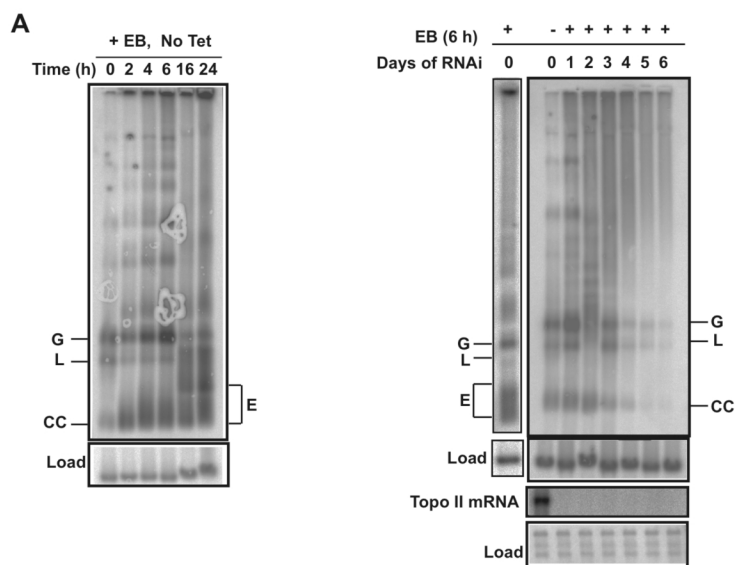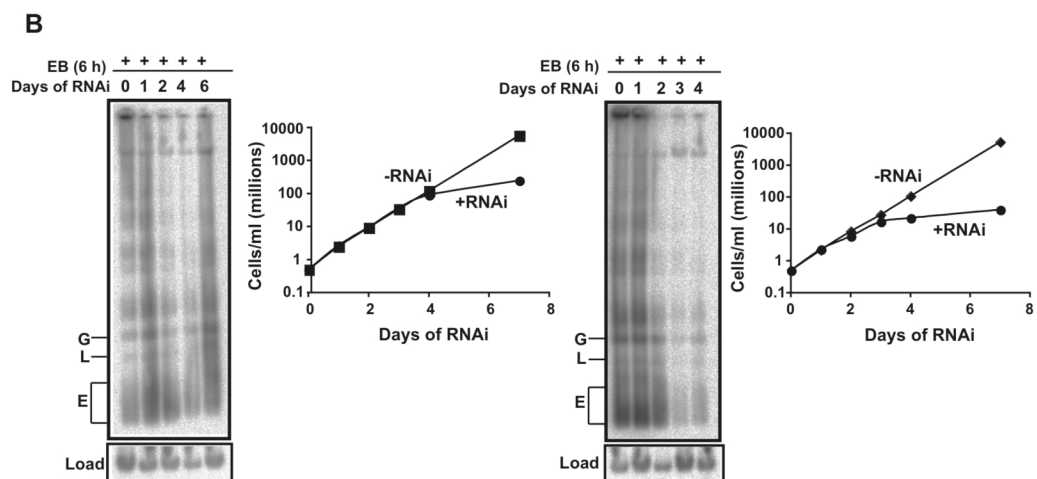

Supplement: Figure S2 — TbTopoIImt is the enzyme that decreases the linking number of EB-bound free minicircles in vivo. We investigated which of the 3 known mitochondrial topoisomerases, TbTopoIImt [29], TbTopoIAmt [30], or TbTopoIB (functional in both nucleus and mitochondrion [31]) was responsible for reducing the linking number of EB-bound free minicircles. In this experiment we used procyclic parasites (this lifecycle stage dwells in the tsetse vector's midgut and is conveniently cultured in the laboratory) as RNAi of these 3 enzymes had been well characterized in that stage [49], [30], [31]. (A) RNAi of TbTopoIImt. Like EB-treated BSF forms, procyclic cell line 29–13 carrying the TbTopoIImt stem-loop RNAi vector but not induced by tetracycline, was susceptible to EB (2 µg/ml) and accumulated fraction E over the 24 h time course (left panel). Also, the effect of TbTopoIImt RNAi on free minicircles was similar to that reported previously [49] (not shown). Furthermore, a northern blot confirmed that RNAi caused a nearly complete loss of the TbTopoIImt mRNA (right panel, next to bottom). For the northern blot, purified total RNA was fractionated on a 1.5% agarose−7% formaldehyde gel [49] and a blot was probed for TbTopoIImt mRNA. The critical experiment is in the main right panel. In this experiment, RNAi of TbTopoIImt was induced by 1 µg/ml tetracycline. Each day, EB (2 µg/ml), was added to culture for 6 h (at day 0, EB was added at the time of induction with tetracycline). Left-most lane shows that EB causes accumulation of fraction E in absence of RNAi. The rest of the right panel showed that knockdown of TbTopoIImt blocked appearance of fraction E. (B) RNAi of TbTopoIAmt (left panel) and TbTopoIB (right panel). The experimental strategy was the same as in Panel A and the efficacy of RNAi was shown by the effect on the growth curves, which were comparable to published results [30], [31]. In control experiments (not shown), EB (2 µg/ml, 6 h) added to uninduced cells led to production o [file ppat.1001226.s002.pdf]

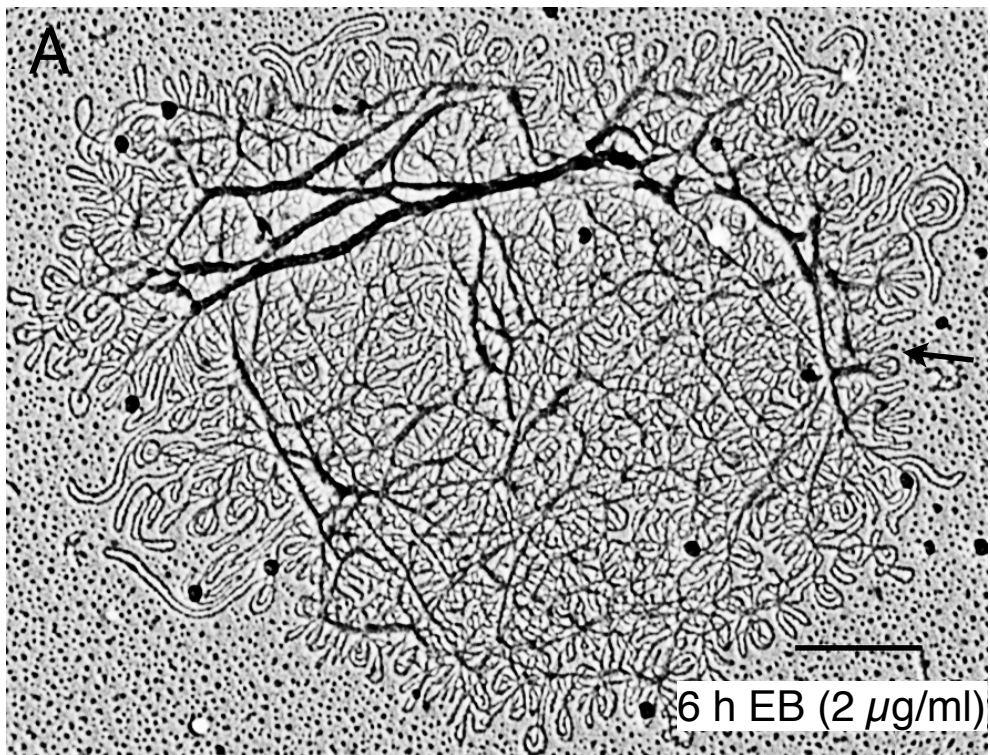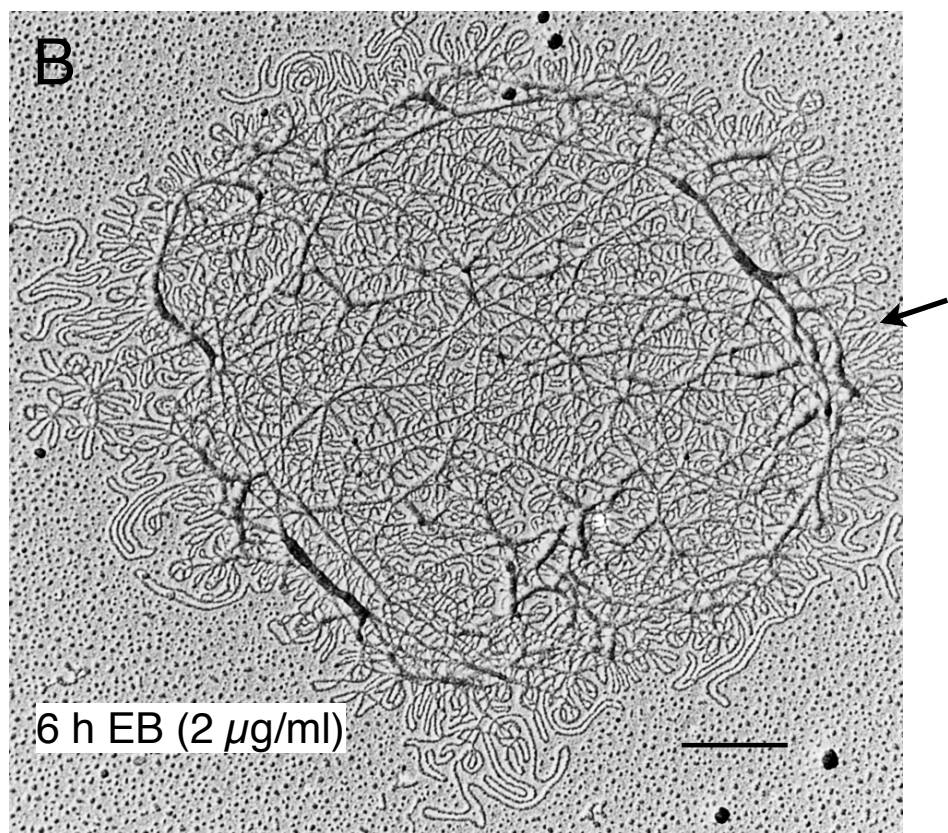

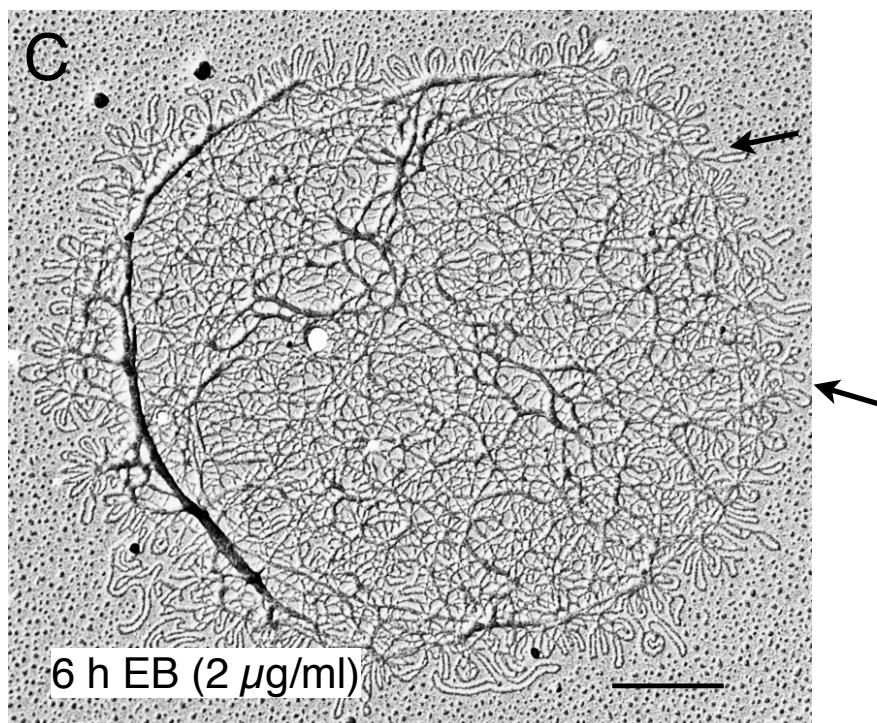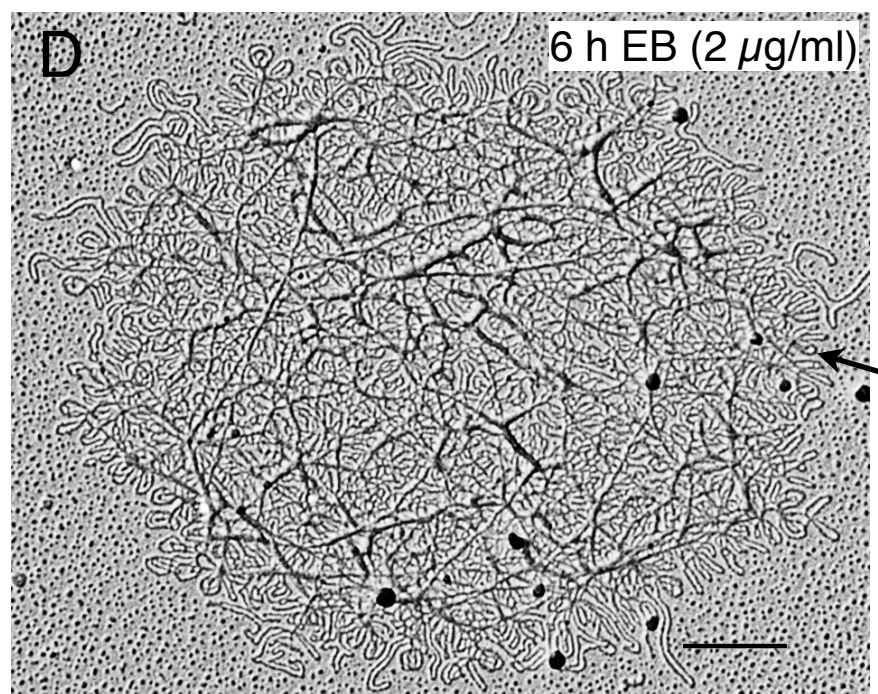

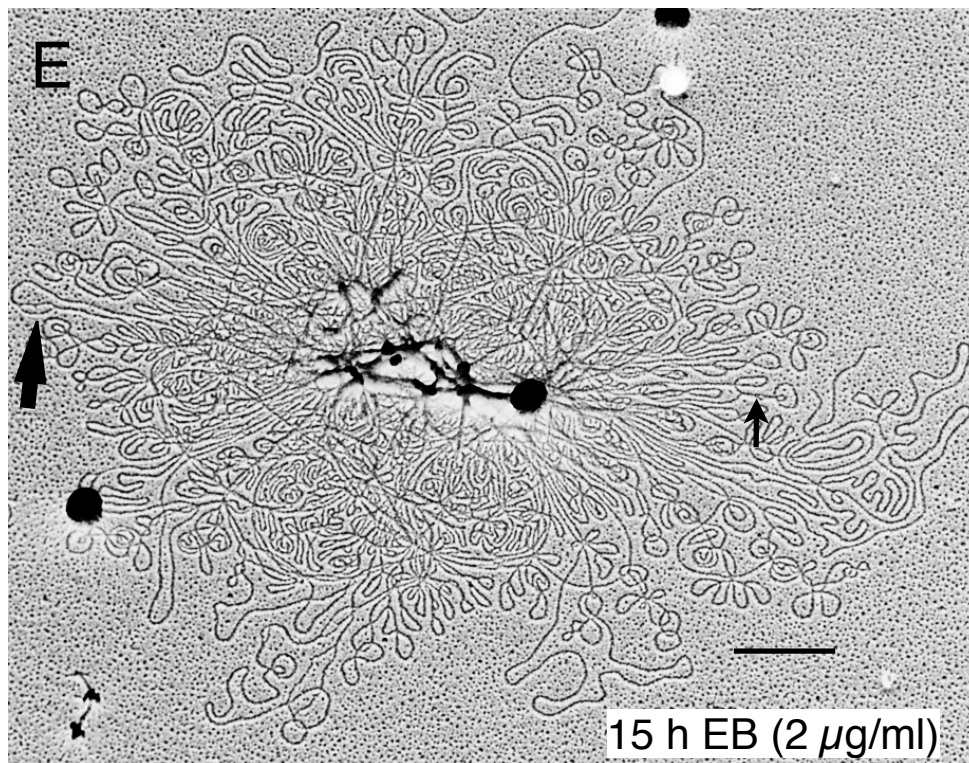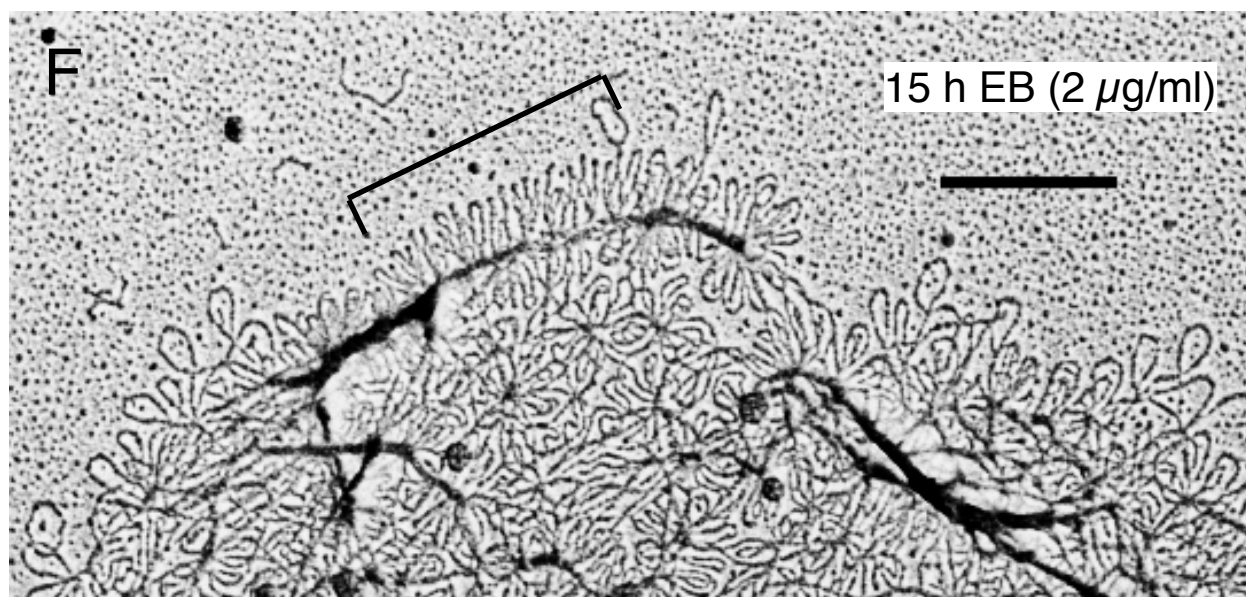

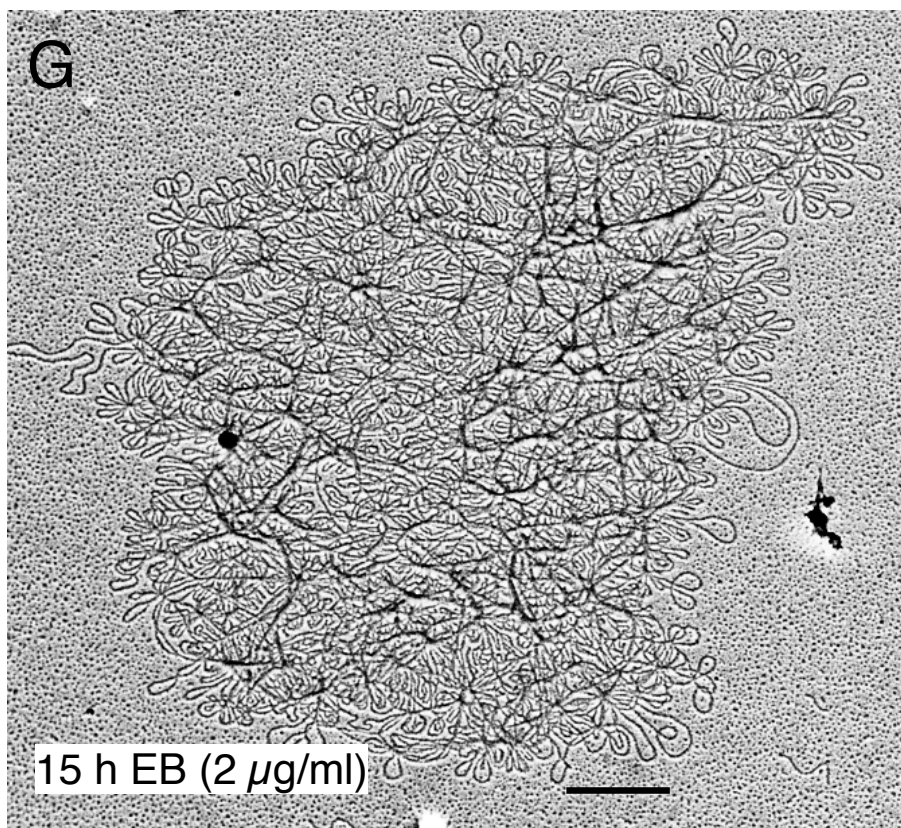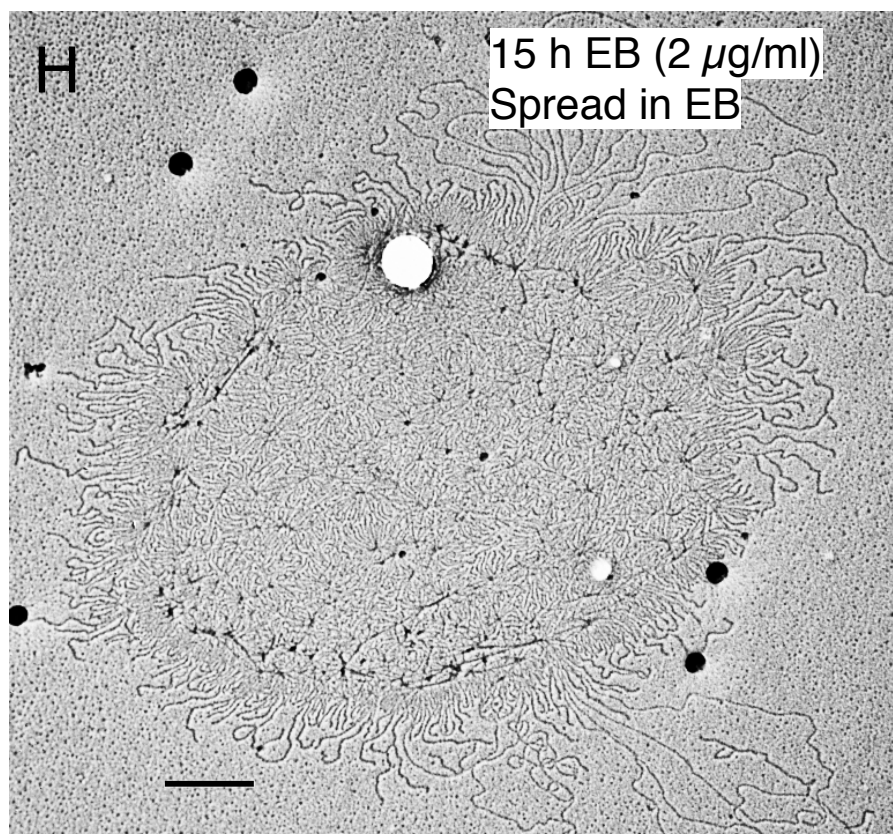

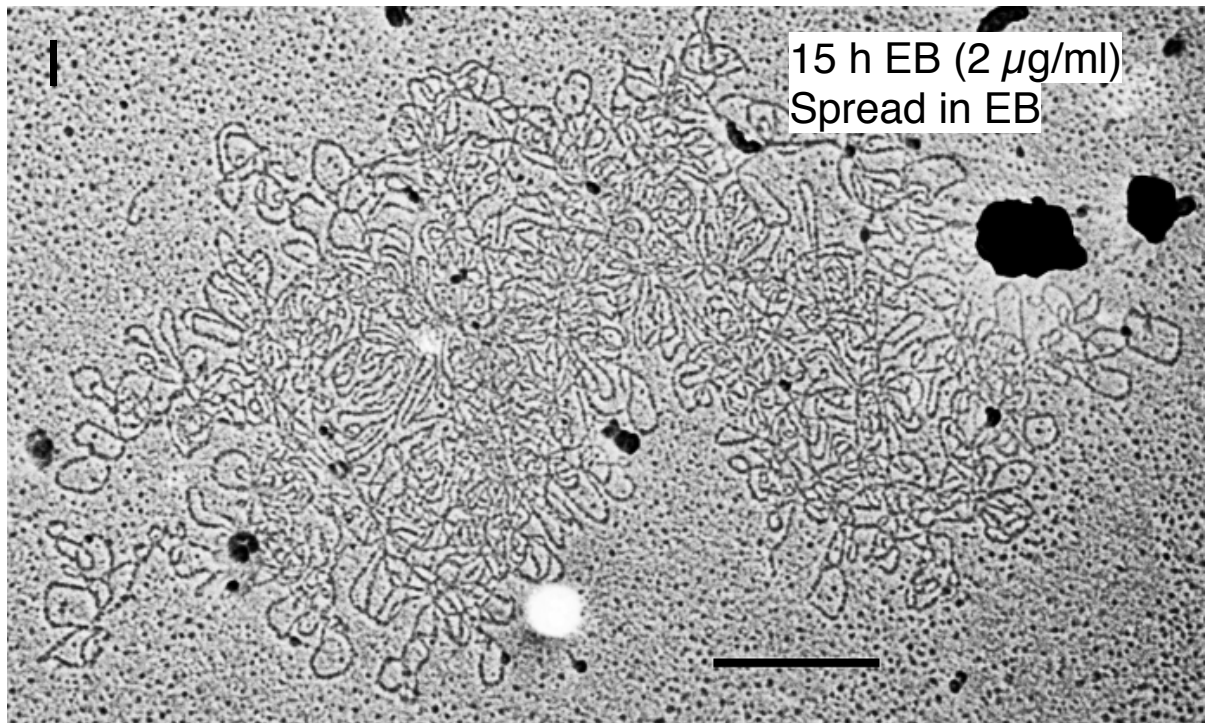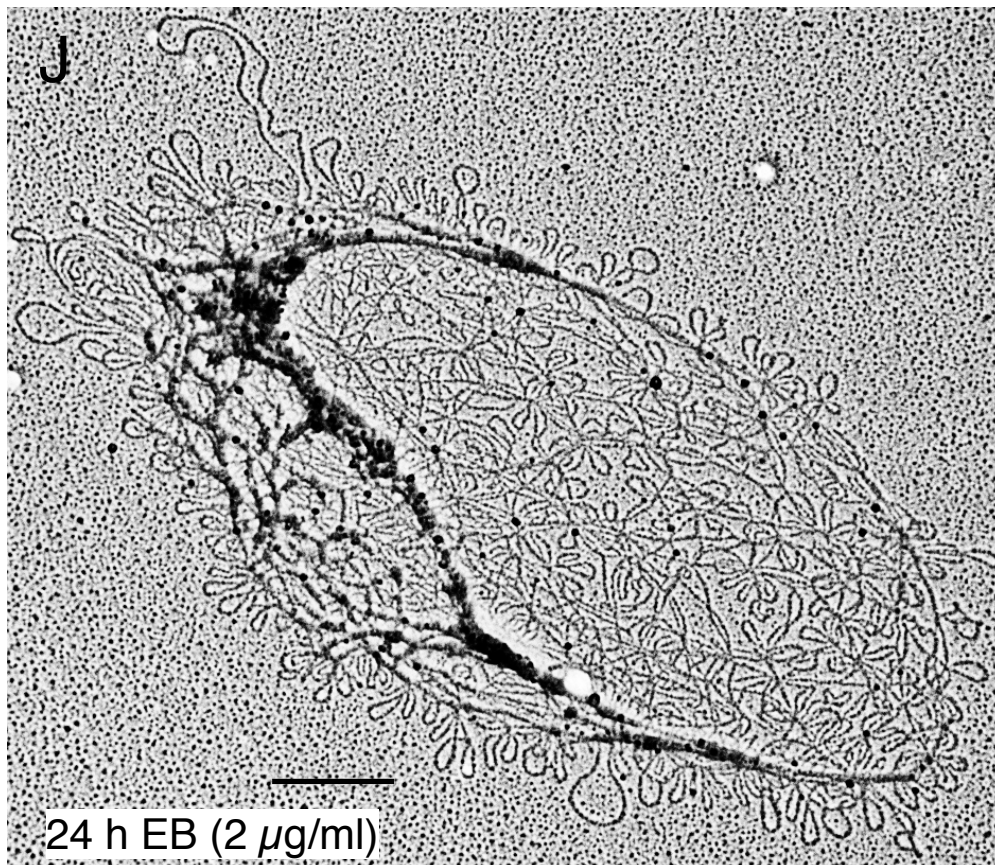

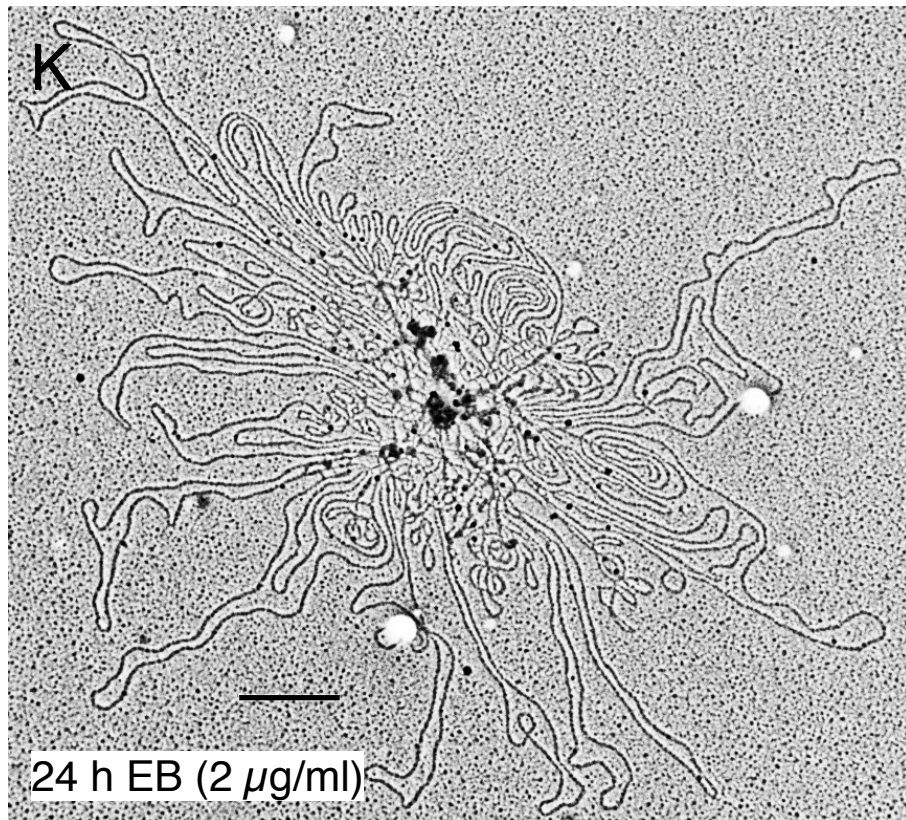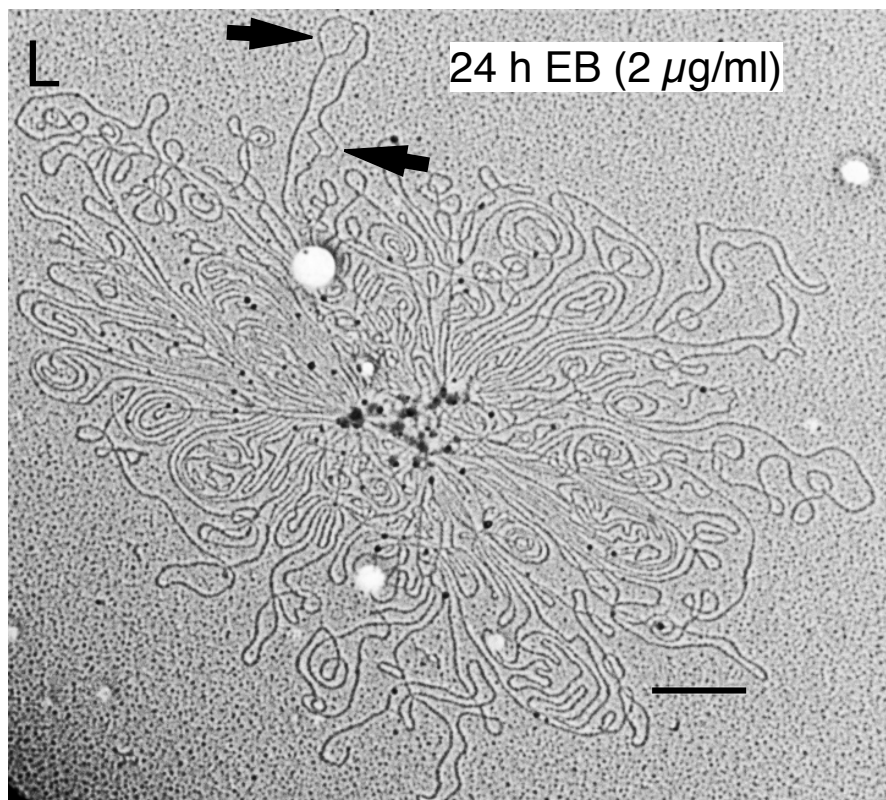

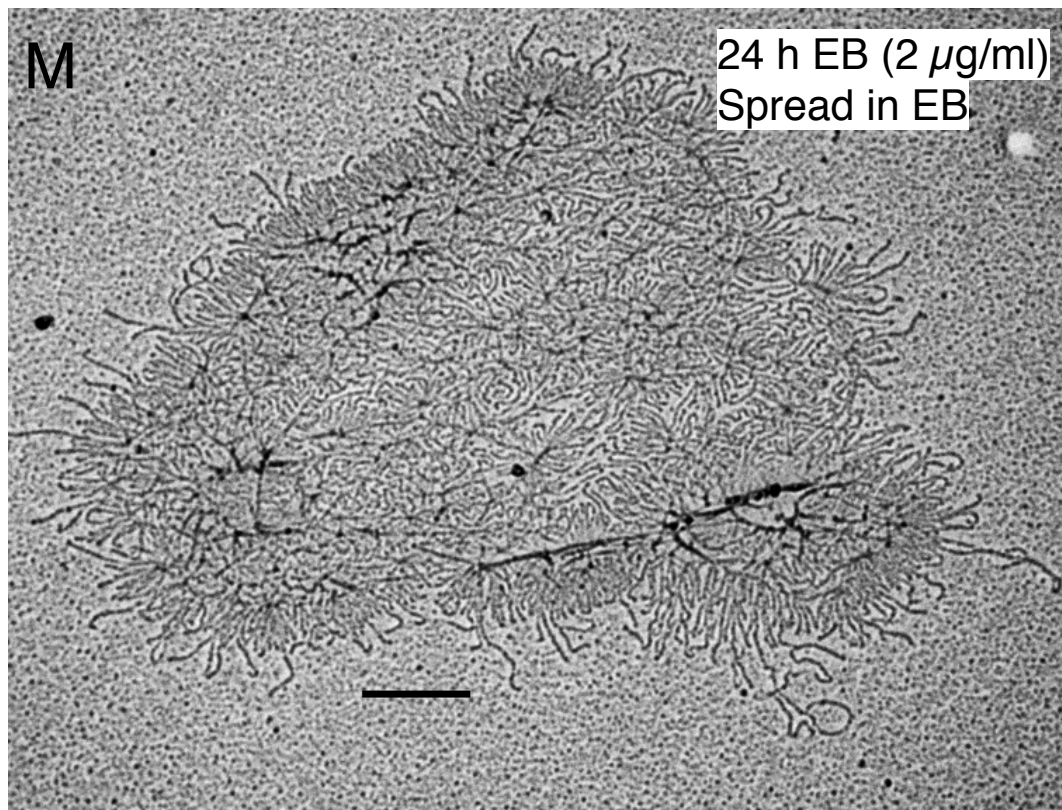

Supplement: Figure S3 — EMs of kDNA networks isolated from cells treated with 2 µg/ml EB. Networks A–D were treated in cells for 6 h, networks E–I were treated in cells for 15 h, and networks J–M were treated in cells for 24 h. Unless otherwise indicated, EB was removed prior to EM. Large loops extending from network edge are maxicircles. Small loops are minicircles. Most of the latter are relaxed, but a few, some of which are marked by arrows, are supertwisted. In crowded networks, especially those present after short treatments with EB, it is usually impossible to determine whether minicircles in the network interior are twisted. Network F has a region on the periphery (spanned by the bracket) in which many neighboring minicircles are twisted. Large arrows in panels E and L indicate maxicircle bubbles. Because the strands within the bubble are thinner than the surrounding strands, the bubbles are probably not due to replication but instead are due to denaturation caused by the high formamide concentrations (up to 40%) during spreading for EM. This is not surprising because maxicircles have AT contents of 76.7%. The presence of two bubbles in a maxicircle in panel D provides another strong argument against the possibility that these bubbles are caused by replication. Networks H, I, and M were spread in the presence of 100 µg/ml EB. Most networks in these preparations resemble networks H and M, having covalently-closed minicircles that become supertwisted by EB in vitro. In contrast, a small number, like network I, have most minicircles relaxed, indicating that they contain nicks or gaps; these networks are further evaluated in Fig. 6. In networks from cells treated with EB for 24 h (networks J–M), some had mostly minicircles (J and M) and some had mostly maxicircles (K and L). Analysis of 24 randomly-selected networks from this preparation indicated that 4 were rich in maxicircles, 11 were rich in minicircles, and 9 were intermediate. Scale bars, 0.5 µm. (6.36 MB PDF) [file ppat.1001226.s003.pdf]

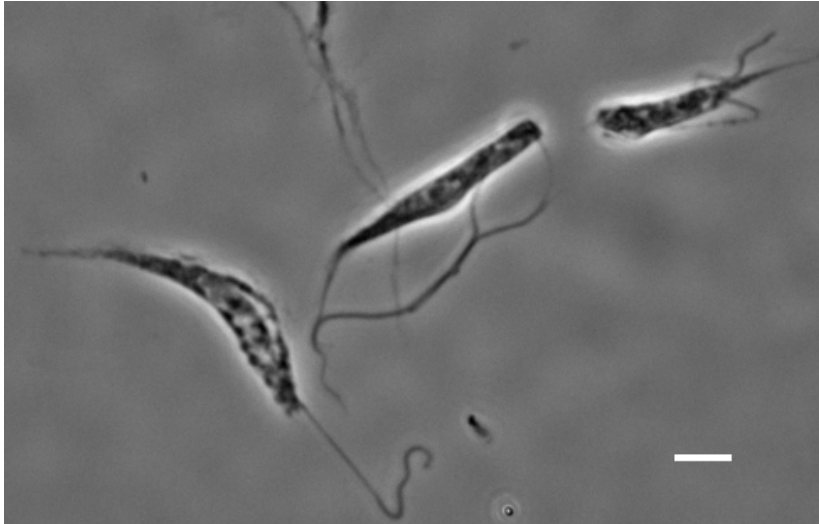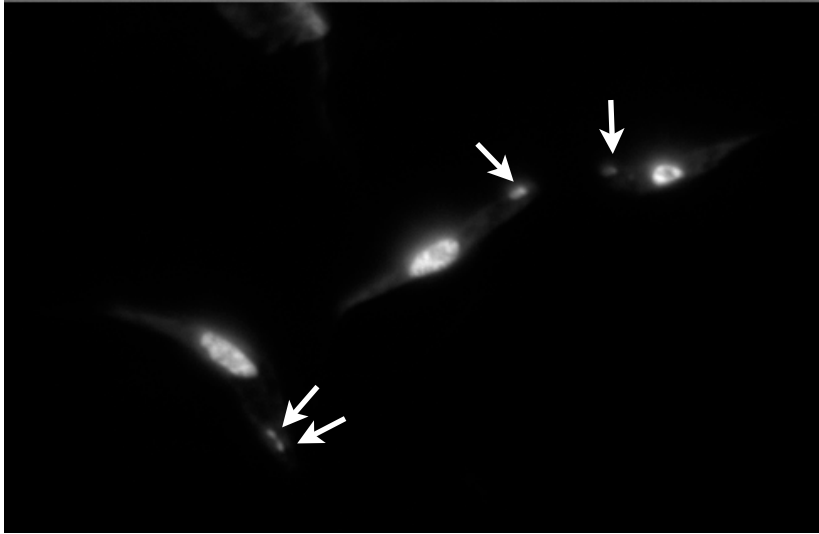

Supplement: Figure S4 — EB staining of T. brucei BSF cells. Log-phase cells were fixed with 4% formaldehyde, washed with PBS-glycine, stained with 2 µg/ml EB for 1 min, and then examined by phase (upper panel) and fluorescence (lower panel) microscopy. Scale bar, 2 µm. Arrows point to kinetoplasts weakly stained by EB. The kinetoplast in the lower left is undergoing division. The larger EB-staining structures are nuclei. (0.23 MB PDF) [file ppat.1001226.s004.pdf]
